# Supplementary material for: African elephant poaching rates correlate with local poverty, national corruption and global ivory price
Source: Nat Commun. 2019 May 28;10:2242. doi: 10.1038/s41467-019-09993-2 (PMC6538616; doi:10.1038/s41467-019-09993-2)
Supplement: Supplementary file 1 — Supplementary Information [file 41467_2019_9993_MOESM1_ESM.pdf]

SUPPLEMENTARY INFORMATION

AFRICAN ELEPHANT POACHING RATES CORRELATE WITH LOCAL POVERTY, NATIONAL CORRUPTION AND GLOBAL IVORY PRICE

HAUENSTEIN ET AL.

Supplementary Tables

Supplementary Table 1: Proxies for supply and demand variables used as covariates in the analysis.

**Covariate**	**Proxy**	**Resolution**	**Source**
Infant mortality rate (IMR)	Number of deaths of children under one year of age per 1,000 live births	Annual, site (interpolation of two datasets)	SEDAC / CIESIN[1], UNICEF[2]
Precipitation (Precip)	Mean annual precipitation in MIKE site in mm	Annual, site	CHIRPS[3]
Corruption perceptions index (CPI)	Expert and businesspeople estimate of perceived public sector corruption	Annual, country	Transparency International[4]
Poverty density (PovDens)	Number of people per km^2 earning less than US$ 1.25 per day	Single measure (2005), site	HarvestChoice[5]
Site area (Area)	Surface area of MIKE site in km^2	Single measure, site	MIKE[6]
Law enforcement adequacy (LawEnf)	Expert estimate of the adequacy of law enforcement provision	Single measure, site	MIKE[6]
Large-scale ivory seizures (Seizures)	Weight in tons of large scale ivory seizures ($>$ 500 kg)	Annual, global	Elephant Trade Information System (ETIS)[7,8]
Ivory price (IvoryPrice)	Average import price of legal mammoth ivory to China, Hong Kong and Macao in US$ kg^{-1}	Annual, global	UN Comtrade Database[9]

**Parameter**	μ	σ	**2.5 %**	**25 %**	**50 %**	**75 %**	**97.5 %**	$\hat{R}$
Int	0.269	0.240	-0.231	0.122	0.272	0.425	0.727	1.033
Precip	0.258	0.067	0.129	0.212	0.259	0.303	0.389	1.001
IMR	-0.092	0.157	-0.415	-0.194	-0.085	0.012	0.214	1.004
CPI	-0.733	0.094	-0.914	-0.798	-0.731	-0.670	-0.544	1.001
PovDens	0.244	0.205	-0.133	0.103	0.235	0.375	0.673	1.008
LawEnf	-0.261	0.210	-0.695	-0.397	-0.255	-0.112	0.119	1.002
Area	-0.020	0.175	-0.364	-0.131	-0.019	0.087	0.342	1.004
Seizures	0.161	0.108	-0.053	0.090	0.160	0.227	0.385	1.005
IvoryPrice	0.485	0.116	0.250	0.407	0.488	0.564	0.710	1.004
λ	1.665	0.427	0.900	1.369	1.651	1.940	2.566	1.002
σ_{site}	1.332	0.193	0.992	1.197	1.318	1.455	1.749	1.002
σ_{year}	0.342	0.079	0.223	0.287	0.328	0.383	0.537	1.003
σ_{country}	0.477	0.313	0.017	0.225	0.445	0.686	1.160	1.026

Supplementary Table 2: Summary table for the model fitted to all data (2002-17) including posterior distribution statistics and $\hat{R}$ for regression coefficients (intercept(Int), and the estimated effects for precipitation(Precip), infant mortality rate (IMR), corruption perception index (CPI), poverty density (PovDens), law enforcement adequacy (LawEnf), site area (Area), large-scale ivory seizures (Seizures) and ivory prices (IvoryPrice)), regularisation parameter λ and the standard deviations of the year-, country- and site-level random effects σ_{site}, σ_{year} and σ_{country}. All covariates were z-transformed and thus all estimated coefficients are directly comparable.

**Parameter**	μ	σ	**2.5 %**	**25 %**	**50 %**	**75 %**	**97.5 %**	$\hat{R}$
Int	0.263	0.253	-0.239	0.097	0.266	0.423	0.777	1.004
Precip	0.250	0.068	0.117	0.206	0.249	0.295	0.384	1.001
IMR	-0.104	0.149	-0.428	-0.193	-0.088	-0.005	0.158	1.006
CPI	-0.713	0.093	-0.897	-0.776	-0.714	-0.651	-0.531	1.001
PovDens	0.179	0.193	-0.156	0.041	0.165	0.303	0.603	1.005
LawEnf	-0.210	0.207	-0.657	-0.339	-0.193	-0.060	0.143	1.007
Area	-0.004	0.151	-0.324	-0.093	0.000	0.091	0.294	1.005
Seizures	0.145	0.104	-0.047	0.075	0.142	0.214	0.363	1.003
IvoryPrice	0.469	0.123	0.207	0.393	0.473	0.553	0.694	1.002
λ	4.025	1.503	1.747	2.947	3.803	4.844	7.660	1.001
σ_{site}	1.356	0.205	1.009	1.211	1.335	1.488	1.804	1.003
σ_{year}	0.354	0.084	0.228	0.295	0.340	0.396	0.552	1.001
$\sigma_{country}$	0.563	0.334	0.04	0.301	0.536	0.784	1.262	1.008

Supplementary Table 3: Summary table as in Supplementary Table 2, but with uniform priors on regularisation parameter λ and random effect standard deviations σ_{site}, σ_{year} and $\sigma_{country}$. The table shows posterior distribution statistics and $\hat{R}$ for regression coefficients (intercept(Int), and the estimated effects for precipitation(Precip), infant mortality rate (IMR), corruption perception index (CPI), poverty density (PovDens), law enforcement adequacy (LawEnf), site area (Area), large-scale ivory seizures (Seizures) and ivory prices (IvoryPrice)), regularisation parameter λ and the standard deviations of the year-, country- and site-level random effects σ_{site}, σ_{year} and $\sigma_{country}$. All covariates were z-transformed and thus all estimated coefficients are directly comparable.

**Parameter**	μ	σ	**2.5 %**	**25 %**	**50 %**	**75 %**	**97.5 %**	$\hat{R}$
Int	0.276	0.238	-0.181	0.121	0.270	0.432	0.749	1.005
Precip	0.261	0.066	0.131	0.218	0.262	0.305	0.392	1.001
CPI	-0.734	0.091	-0.915	-0.796	-0.732	-0.673	-0.558	1.001
PovDens	0.256	0.213	-0.165	0.111	0.255	0.397	0.681	1.015
LawEnf	-0.239	0.211	-0.669	-0.374	-0.232	-0.091	0.141	1.001
Area	-0.001	0.176	-0.367	-0.111	0.000	0.110	0.346	1.006
Seizures	0.256	0.213	-0.165	0.111	0.255	0.397	0.681	1.015
IvoryPrice	0.560	0.104	0.346	0.494	0.561	0.628	0.755	1.006

Supplementary Table 4: Summary table as in Supplementary Table 2, but with IMR removed from the model. The table shows posterior distribution statistics and $\hat{R}$ for regression coefficients (intercept(Int), and the estimated effects for precipitation(Precip), infant mortality rate (IMR), corruption perception index (CPI), poverty density (PovDens), law enforcement adequacy (LawEnf), site area (Area), large-scale ivory seizures (Seizures) and ivory prices (IvoryPrice)). All covariates were z-transformed and thus all estimated coefficients are directly comparable.

**Parameter**	μ	σ	**2.5 %**	**25 %**	**50 %**	**75 %**	**97.5 %**	$\hat{R}$
Int	0.282	0.211	-0.141	0.149	0.283	0.419	0.688	1.006
Precip	0.250	0.066	0.122	0.208	0.250	0.293	0.384	1.001
IMR	0.624	0.216	0.189	0.485	0.632	0.773	1.040	1.011
CPI	-0.725	0.088	-0.891	-0.784	-0.726	-0.666	-0.550	1.002
PovDens	0.120	0.186	-0.218	-0.008	0.109	0.239	0.503	1.004
LawEnf	-0.078	0.191	-0.472	-0.197	-0.071	0.045	0.298	1.008
Area	-0.027	0.171	-0.378	-0.131	-0.027	0.082	0.306	1.006
Seizures	0.125	0.102	-0.065	0.059	0.122	0.191	0.330	1.001
IvoryPrice	0.557	0.104	0.345	0.493	0.559	0.624	0.764	1.004

Supplementary Table 5: Summary table as in Supplementary Table 2, but with IMR only varying by site and not with time. The table shows posterior distribution statistics and $\hat{R}$ for regression coefficients (intercept(Int), and the estimated effects for precipitation(Precip), infant mortality rate (IMR), corruption perception index (CPI), poverty density (PovDens), law enforcement adequacy (LawEnf), site area (Area), large-scale ivory seizures (Seizures) and ivory prices (IvoryPrice)). All covariates were z-transformed and thus all estimated coefficients are directly comparable.

**Parameter**	μ	σ	**2.5 %**	**25 %**	**50 %**	**75 %**	**97.5 %**	$\hat{R}$
Int	0.272	0.253	-0.214	0.102	0.271	0.437	0.792	1.002
Precip	0.261	0.067	0.132	0.214	0.260	0.306	0.395	1.001
IMR	-0.121	0.168	-0.473	-0.233	-0.111	-0.005	0.195	1.012
CPI	-0.725	0.092	-0.910	-0.787	-0.724	-0.662	-0.550	1.002
PovDens	0.257	0.217	-0.161	0.105	0.254	0.408	0.672	1.007
LawEnf	-0.283	0.219	-0.734	-0.425	-0.277	-0.133	0.120	1.007
Area	-0.002	0.186	-0.356	-0.123	-0.006	0.109	0.383	1.011
Seizures_{-1}	-0.053	0.147	-0.335	-0.147	-0.057	0.035	0.266	1.004
IvoryPrice	0.606	0.169	0.247	0.501	0.611	0.722	0.921	1.004

Supplementary Table 6: Summary table as in Supplementary Table 2, but with a 1-year lag in large-scale ivory seizures. The table shows posterior distribution statistics and $\hat{R}$ for regression coefficients (intercept (i)nt), and the estimated effects for precipitation (Precip), infant mortality rate (IMR), corruption perception index (CPI), poverty density (PovDens), law enforcement adequacy (LawEnf), site area (Area), 1-year lagged large-scale ivory seizures (Seizures_{-1}) and ivory prices (IvoryPrice)). All covariates were z-transformed and thus all estimated coefficients are directly comparable.

**Parameter**	μ	σ	**2.5 %**	**25 %**	**50 %**	**75 %**	**97.5 %**	$\hat{R}$
Int	0.275	0.261	-0.226	0.100	0.283	0.447	0.797	1.007
Precip	0.260	0.066	0.127	0.215	0.260	0.305	0.386	1.002
IMR	-0.115	0.165	-0.460	-0.223	-0.104	-0.002	0.189	1.008
CPI	-0.732	0.093	-0.919	-0.791	-0.731	-0.668	-0.551	1.001
PovDens	0.254	0.215	-0.142	0.114	0.248	0.389	0.694	1.005
LawEnf	-0.269	0.222	-0.723	-0.415	-0.262	-0.112	0.142	1.002
Area	-0.016	0.203	-0.433	-0.142	-0.012	0.114	0.384	1.006
Seizures$_{-2}$	0.125	0.108	-0.079	0.051	0.120	0.193	0.349	1.005
IvoryPrice	0.497	0.122	0.250	0.418	0.499	0.575	0.733	1.004

Supplementary Table 7: Summary table as in Supplementary Table 2, but with a 2-year lag in large-scale ivory seizures. The table shows posterior distribution statistics and $\hat{R}$ for regression coefficients (intercept (i)nt), and the estimated effects for precipitation (Precip), infant mortality rate (IMR), corruption perception index (CPI), poverty density (PovDens), law enforcement adequacy (LawEnf), site area (Area), 2-year lagged large-scale ivory seizures (Seizures$_{-2}$) and ivory prices (IvoryPrice)). All covariates were z-transformed and thus all estimated coefficients are directly comparable.

**Parameter**	μ	σ	**2.5 %**	**25 %**	**50 %**	**75 %**	**97.5 %**	$\hat{R}$
Int	0.294	0.277	-0.223	0.110	0.285	0.472	0.842	1.008
Precip	0.257	0.068	0.121	0.210	0.257	0.302	0.388	1.001
IMR	-0.160	0.177	-0.525	-0.275	-0.149	-0.034	0.171	1.003
CPI	-0.716	0.093	-0.902	-0.779	-0.715	-0.652	-0.538	1.001
PovDens	0.243	0.219	-0.170	0.090	0.234	0.384	0.701	1.006
LawEnf	-0.278	0.220	-0.742	-0.420	-0.267	-0.125	0.116	1.005
Area	0.003	0.195	-0.390	-0.123	0.002	0.129	0.388	1.012
Seizures	0.163	0.146	-0.109	0.066	0.157	0.258	0.459	1.002
IvoryPrice$_{-1}$	0.377	0.161	0.062	0.267	0.377	0.485	0.694	1.003

Supplementary Table 8: Summary table as in Supplementary Table 2, but with a 1-year lag in ivory prices. The table shows posterior distribution statistics and $\hat{R}$ for regression coefficients (intercept (i)nt), and the estimated effects for precipitation (Precip), infant mortality rate (IMR), corruption perception index (CPI), poverty density (PovDens), law enforcement adequacy (LawEnf), site area (Area), large-scale ivory seizures (Seizures) and 1-year lagged ivory prices (IvoryPrice$_{-1}$)). All covariates were z-transformed and thus all estimated coefficients are directly comparable.

**Parameter**	μ	σ	**2.5 %**	**25 %**	**50 %**	**75 %**	**97.5 %**	$\hat{R}$
Int	0.290	0.256	-0.194	0.113	0.281	0.457	0.819	1.003
Precip	0.253	0.066	0.123	0.207	0.253	0.298	0.379	1.002
IMR	-0.179	0.183	-0.560	-0.301	-0.170	-0.050	0.151	1.002
CPI	-0.709	0.092	-0.892	-0.771	-0.710	-0.648	-0.525	1.001
PovDens	0.247	0.217	-0.141	0.093	0.234	0.393	0.688	1.001
LawEnf	-0.284	0.230	-0.748	-0.436	-0.274	-0.122	0.126	1.005
Area	-0.005	0.186	-0.398	-0.124	-0.004	0.116	0.361	1.006
Seizures	0.291	0.162	-0.024	0.185	0.290	0.395	0.615	1.001
IvoryPrice$_{-2}$	0.187	0.165	-0.119	0.070	0.185	0.296	0.525	1.001

Supplementary Table 9: Summary table as in Supplementary Table 2, but with a 2-year lag in ivory prices. The table shows posterior distribution statistics and $\hat{R}$ for regression coefficients (intercept (i)nt), and the estimated effects for precipitation (Precip), infant mortality rate (IMR), corruption perception index (CPI), poverty density (PovDens), law enforcement adequacy (LawEnf), site area (Area), large-scale ivory seizures (Seizures) and 2-year lagged ivory prices (IvoryPrice$_{-2}$)). All covariates were z-transformed and thus all estimated coefficients are directly comparable.

**Parameter**	μ	σ	**2.5 %**	**25 %**	**50 %**	**75 %**	**97.5 %**	$\hat{R}$
Int	0.257	0.273	-0.307	0.088	0.257	0.442	0.772	1.010
Precip	0.258	0.066	0.132	0.213	0.258	0.301	0.385	1.001
IMR	-0.153	0.174	-0.523	-0.265	-0.147	-0.033	0.167	1.001
CPI	-0.721	0.093	-0.909	-0.782	-0.719	-0.657	-0.537	1.001
PovDens	0.225	0.221	-0.198	0.069	0.226	0.378	0.659	1.006
LawEnf	-0.288	0.228	-0.750	-0.433	-0.276	-0.136	0.136	1.001
Area	-0.015	0.189	-0.388	-0.136	-0.017	0.105	0.367	1.003
Seizures_{-1}	0.163	0.136	-0.107	0.073	0.157	0.252	0.444	1.009
IvoryPrice_{-1}	0.386	0.153	0.091	0.280	0.386	0.485	0.689	1.006

Supplementary Table 10: Summary table as in Supplementary Table 2, but with 1-year lags in large-scale ivory seizures and ivory prices. The table shows posterior distribution statistics and $\hat{R}$ for regression coefficients (intercept (i)nt), and the estimated effects for precipitation (Precip), infant mortality rate (IMR), corruption perception index (CPI), poverty density (PovDens), law enforcement adequacy (LawEnf), site area (Area), 1-year lagged large-scale ivory seizures (Seizures_{-1}) and 1-year lagged ivory prices (IvoryPrice_{-1})). All covariates were z-transformed and thus all estimated coefficients are directly comparable.

**Parameter**	μ	σ	**2.5 %**	**25 %**	**50 %**	**75 %**	**97.5 %**	$\hat{R}$
Int	0.292	0.259	-0.241	0.128	0.293	0.461	0.796	1.002
Precip	0.259	0.067	0.129	0.214	0.259	0.303	0.390	1.001
IMR	-0.144	0.178	-0.516	-0.259	-0.136	-0.020	0.180	1.005
CPI	-0.715	0.092	-0.896	-0.778	-0.713	-0.653	-0.531	1.001
PovDens	0.240	0.208	-0.155	0.095	0.235	0.382	0.654	1.008
LawEnf	-0.283	0.220	-0.717	-0.425	-0.280	-0.125	0.120	1.007
Area	0.003	0.189	-0.373	-0.116	0.002	0.119	0.396	1.011
Seizures_{-2}	0.068	0.179	-0.282	-0.047	0.059	0.174	0.441	1.003
IvoryPrice_{-1}	0.432	0.193	0.052	0.307	0.432	0.560	0.822	1.002

Supplementary Table 11: Summary table as in Supplementary Table 2, but with a 2-year lag in large-scale ivory seizures and a 1-year lag in ivory prices. The table shows posterior distribution statistics and $\hat{R}$ for regression coefficients (intercept (i)nt), and the estimated effects for precipitation (Precip), infant mortality rate (IMR), corruption perception index (CPI), poverty density (PovDens), law enforcement adequacy (LawEnf), site area (Area), 2-year lagged large-scale ivory seizures (Seizures_{-2}) and 1-year lagged ivory prices (IvoryPrice_{-1})). All covariates were z-transformed and thus all estimated coefficients are directly comparable.

**Parameter**	μ	σ	**2.5 %**	**25 %**	**50 %**	**75 %**	**97.5 %**	$\hat{R}$
Int	0.273	0.276	-0.243	0.088	0.258	0.456	0.839	1.002
Precip	0.254	0.067	0.125	0.210	0.254	0.298	0.387	1.001
IMR	-0.195	0.194	-0.596	-0.325	-0.186	-0.056	0.164	1.007
CPI	-0.711	0.094	-0.898	-0.774	-0.713	-0.648	-0.533	1.001
PovDens	0.232	0.211	-0.173	0.095	0.225	0.365	0.656	1.004
LawEnf	-0.315	0.226	-0.792	-0.458	-0.304	-0.156	0.099	1.004
Area	-0.030	0.191	-0.416	-0.154	-0.025	0.092	0.361	1.014
Seizures_{-1}	0.245	0.180	-0.090	0.120	0.239	0.360	0.616	1.007
IvoryPrice_{-2}	0.193	0.182	-0.141	0.070	0.185	0.308	0.573	1.013

Supplementary Table 12: Summary table as in Supplementary Table 2, but with a 1-year lag in large-scale ivory seizures and a 2-year lag in ivory prices. The table shows posterior distribution statistics and $\hat{R}$ for regression coefficients (intercept (i)nt), and the estimated effects for precipitation (Precip), infant mortality rate (IMR), corruption perception index (CPI), poverty density (PovDens), law enforcement adequacy (LawEnf), site area (Area), 1-year lagged large-scale ivory seizures (Seizures_{-1}) and 2-year lagged ivory prices (IvoryPrice_{-2})). All covariates were z-transformed and thus all estimated coefficients are directly comparable.

**Parameter**	μ	σ	**2.5 %**	**25 %**	**50 %**	**75 %**	**97.5 %**	$\hat{R}$
Int	0.295	0.276	-0.244	0.111	0.292	0.469	0.857	1.005
Precip	0.255	0.066	0.123	0.212	0.256	0.299	0.380	1.003
IMR	-0.181	0.191	-0.589	-0.304	-0.173	-0.047	0.169	1.004
CPI	-0.714	0.094	-0.896	-0.780	-0.713	-0.650	-0.534	1.002
PovDens	0.233	0.210	-0.151	0.087	0.227	0.378	0.651	1.021
LawEnf	-0.285	0.224	-0.752	-0.429	-0.272	-0.129	0.115	1.001
Area	0.013	0.183	-0.349	-0.101	0.009	0.128	0.382	1.018
Seizures_{-2}	0.252	0.161	-0.049	0.142	0.246	0.357	0.572	1.004
IvoryPrice_{-2}	0.209	0.169	-0.099	0.090	0.204	0.319	0.545	1.009

Supplementary Table 13: Summary table as in Supplementary Table 2, but with 2-year lags in large-scale ivory seizures and ivory prices. The table shows posterior distribution statistics and $\hat{R}$ for regression coefficients (intercept (i)nt), and the estimated effects for precipitation (Precip), infant mortality rate (IMR), corruption perception index (CPI), poverty density (PovDens), law enforcement adequacy (LawEnf), site area (Area), 2-year lagged large-scale ivory seizures (Seizures_{-2}) and 2-year lagged ivory prices (IvoryPrice_{-2})). All covariates were z-transformed and thus all estimated coefficients are directly comparable.

Supplementary Figures

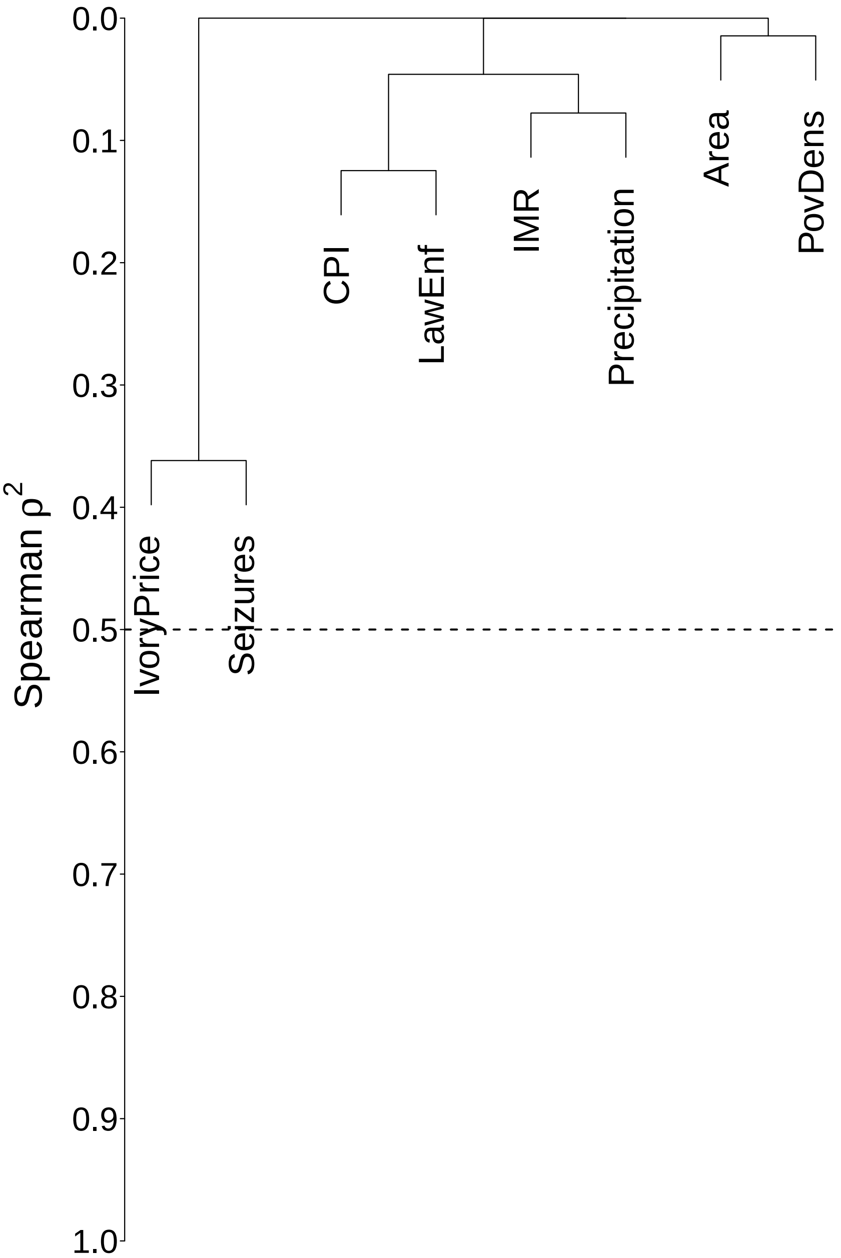

Supplementary Figure 1: Cluster representation to visualise collinearity among covariates. Measure of correlation is Spearman's ρ^2. Dashed line indicates the threshold for a problematic correlation among covariates (Spearman's $\rho^2 > 0.5$).

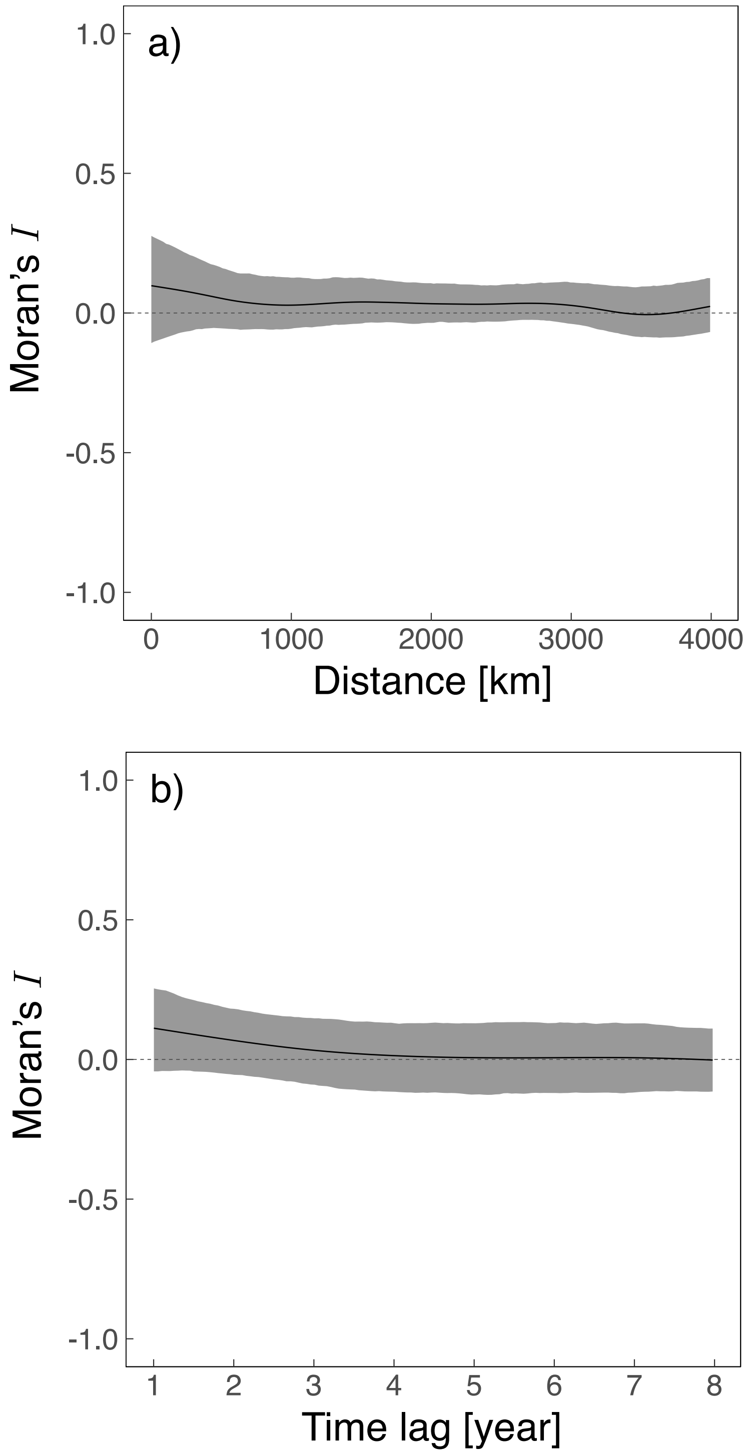

Supplementary Figure 2: Testing for structural dependencies. a) Spatial and b) temporal autocorrelation patterns in model residuals illustrated by estimated Moran similarity against a) spatial distance and b) time lag. The displayed error envelopes represent 90 % confidence intervals derived via bootstrapping.

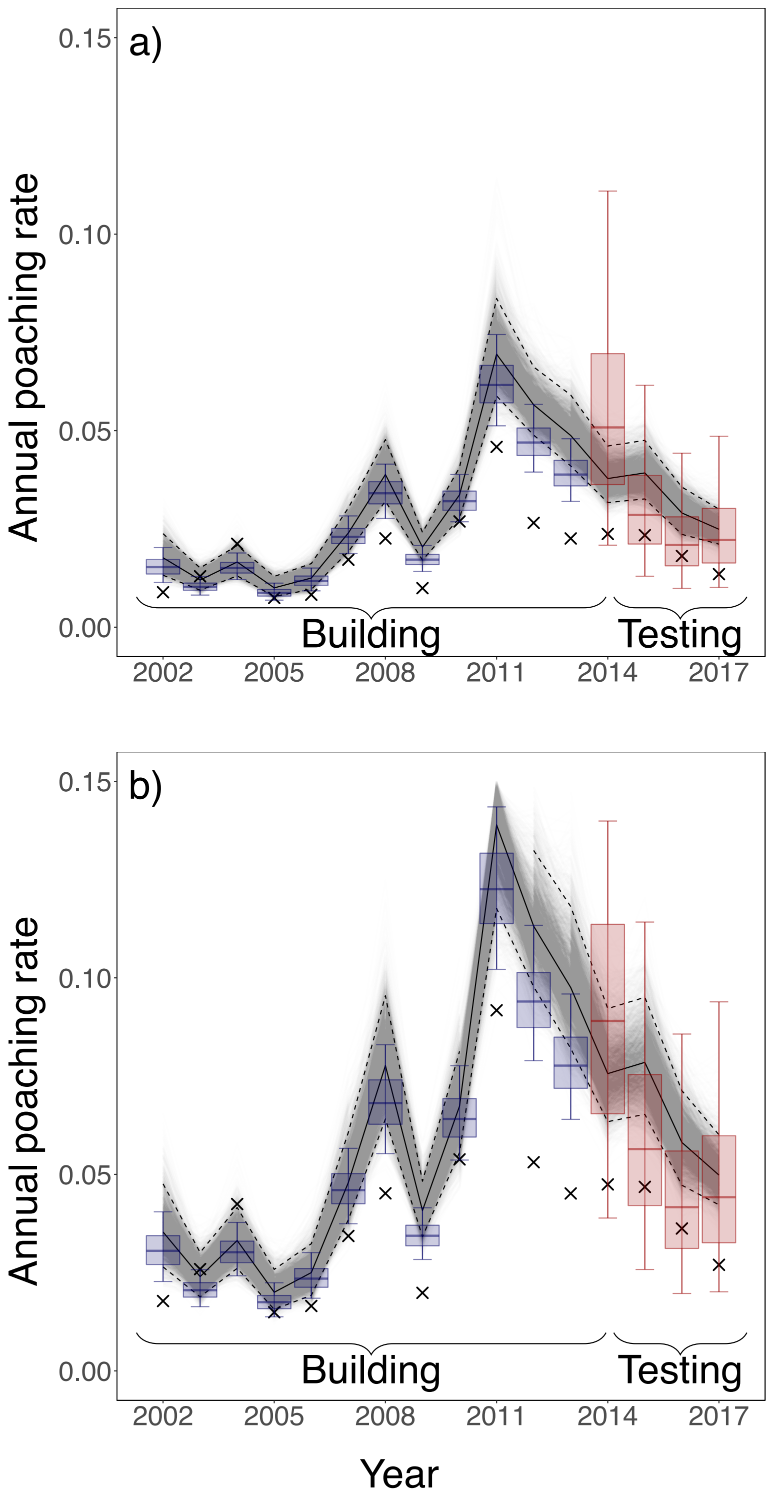

Supplementary Figure 3: Sensitivity of estimated poaching rates to assumed natural mortality rates. a) 2 % and b) 4 % natural morality. Annual estimates of per capita poaching rate across 53 MIKE monitoring sites. Displayed estimates are annual median poaching rates across all sites in each year, derived from 3,000 MCMC samples. Grey lines display estimates from the model fitted to all data, with solid black line showing median estimates and dashed lines outlining 90 % CI. Blue boxes represent estimates from the model (same structure) fitted to training data (2002-13) only, red colour highlights estimates for test data (2014-17). Boxplot centre lines represent median estimates, box bounds first and third quartiles, and whiskers 90 % CIs. Crosses represent overall observed poaching rate across all sites and will be biased towards sites where more carcasses are found.

Supplementary References

[1] Center for International Earth Science Information Network CIESIN Columbia University. Poverty mapping project: Global subnational infant mortality rates. https://doi.org/10.7927/H4PZ56R2 (2018).

[2] UN Children's Fund (UNICEF). Levels & trends in child mortality. http://www.refworld.org/docid/55f6c3304.html [accessed 19 December 2017] (2017).

[3] Funk, C. *et al.* The climate hazards infrared precipitation with stations–a new environmental record for monitoring extremes. *Scientific Data* **2**, 150066 (2015).

[4] Transparency International. Corruption Perceptions Index. http://www.transparency.org/cpi/ [accessed 3 Nov. 2017] (2017).

[5] HarvestChoice. Poverty density $1.25/day (pers./sq. km., circa 2005). *International Food Policy Research Institute, Washington, DC., and University of Minnesota, St. Paul, MN.* http://harvestchoice.org/data/tpov_pd125 [accessed 16 Oct. 2017] (2015).

[6] Monitoring the Illegal Killing of Elephants (MIKE). MIKE data and reports. https://fusiontables.google.com/DataSource?docid=1gMHluFHQyJkokmOT7ux1RSYUOoxPigqz5LE2UulS [accessed 12 September 2017] (2017).

[7] Milliken, T. Progress in the implementation of the Elephant Trade Information System (ETIS). *Pachyderm* 85–90 (2014).

[8] Convention on International Trade in Endangered Species. Status of elephant populations, levels of illegal killing and the trade in ivory: A report to the CITES Standing Committee,. In *17th Meeting of the Conference of the Parties Johannesburg, South Africa*, vol. CoP SC69 Doc. 51.1 Annex. https://cites.org/sites/default/files/eng/prog/MIKE/SC/E-SC69-51-01-A.pdf [accessed 26 Jan. 2018] (2017).

[9] United Nations Statistics Division. UN comtrade. http://comtrade.un.org/ [accessed 30 Sept. 2017] (2018).
