# Supplementary material for: African elephant poaching rates correlate with local poverty, national corruption and global ivory price
Source: Nat Commun. 2019 May 28;10:2242. doi: 10.1038/s41467-019-09993-2 (PMC6538616; doi:10.1038/s41467-019-09993-2)
Supplement: Supplementary file 3 — Reporting Summary [file 41467_2019_9993_MOESM3_ESM.pdf]

nature research

Corresponding author(s): Severin Hauenstein

Last updated by author(s): Apr 11, 2019

Reporting Summary

Nature Research wishes to improve the reproducibility of the work that we publish. This form provides structure for consistency and transparency in reporting. For further information on Nature Research policies, see Authors & Referees and the Editorial Policy Checklist.

Statistics

For all statistical analyses, confirm that the following items are present in the figure legend, table legend, main text, or Methods section.

n/a	Confirmed	
☐	☒	The exact sample size (*n*) for each experimental group/condition, given as a discrete number and unit of measurement
☐	☒	A statement on whether measurements were taken from distinct samples or whether the same sample was measured repeatedly
☐	☒	The statistical test(s) used AND whether they are one- or two-sided<br>*Only common tests should be described solely by name; describe more complex techniques in the Methods section.*
☐	☒	A description of all covariates tested
☐	☒	A description of any assumptions or corrections, such as tests of normality and adjustment for multiple comparisons
☐	☒	A full description of the statistical parameters including central tendency (e.g. means) or other basic estimates (e.g. regression coefficient) AND variation (e.g. standard deviation) or associated estimates of uncertainty (e.g. confidence intervals)
☒	☐	For null hypothesis testing, the test statistic (e.g. *F*, *t*, *r*) with confidence intervals, effect sizes, degrees of freedom and *P* value noted<br>*Give P values as exact values whenever suitable.*
☐	☒	For Bayesian analysis, information on the choice of priors and Markov chain Monte Carlo settings
☐	☒	For hierarchical and complex designs, identification of the appropriate level for tests and full reporting of outcomes
☐	☒	Estimates of effect sizes (e.g. Cohen's *d*, Pearson's *r*), indicating how they were calculated

Our web collection on statistics for biologists contains articles on many of the points above.

Software and code

Policy information about availability of computer code

Data collection	All analysis and data preparation carried out in R (version 3.3). Code and data provided in a figshare repository.
Data analysis	All analysis and data preparation carried out in R (version 3.3). Code and data provided in a figshare repository.

For manuscripts utilizing custom algorithms or software that are central to the research but not yet described in published literature, software must be made available to editors/reviewers. We strongly encourage code deposition in a community repository (e.g. GitHub). See the Nature Research guidelines for submitting code & software for further information.

Data

Policy information about availability of data

All manuscripts must include a data availability statement. This statement should provide the following information, where applicable:

- Accession codes, unique identifiers, or web links for publicly available datasets
- A list of figures that have associated raw data
- A description of any restrictions on data availability

Data and R code to reproduce the analysis are available in a figshare data repository at https://doi.org/10.6084/m9.figshare.7713245

Field-specific reporting

Please select the one below that is the best fit for your research. If you are not sure, read the appropriate sections before making your selection.

☐ Life sciences ☐ Behavioural & social sciences ☒ Ecological, evolutionary & environmental sciences

For a reference copy of the document with all sections, see nature.com/documents/nr-reporting-summary-flat.pdf

Ecological, evolutionary & environmental sciences study design

All studies must disclose on these points even when the disclosure is negative.

Study description	Statistical analysis of African elephant carcass-encounter data to identify correlates of elephant poaching and predict local, regional and continental poaching levels. This includes a total of total NN carcasses, collected at NNN unique site / year combinations.
Research sample	Data provided by the 'Monitoring the Illegal Killing of Elephants' (MIKE) programme, which collates annual elephant carcass counts from 53 sites in 29 countries across Africa. These sites together hold > 50% of remaining African Elephants.
Sampling strategy	We included ALL available MIKE data.
Data collection	Data collected by the 'Monitoring the Illegal Killing of Elephants' (MIKE) programme - each site allocates a reporting officer who collects, once per year, the required data from ranger observation records and sends them to the MIKE offices where we collated them.
Timing and spatial scale	Carcass counts reported annually from 2002 to 2017, varying by site. Sites vary in size from 10s to 1000s of square kilometres. As required by CITES, wildlife authorities report once per year.
Data exclusions	No data were excluded.
Reproducibility	At each site, rangers on regular patrols record the location of any elephant carcass encountered and identify whether death was the result of natural mortality, management or illegal killing. All sites have had support from the MIKE office in this. All code has been replicated independently by two of the co-authors, successfully regenerating results.
Randomization	Annual carcass counts are collated by site. Where possible, we collated covariates similarly. To account for structural dependencies, the statistical model includes random intercepts for year, site and country.
Blinding	Not possible for this observational study, where rangers collected data on regular patrols. Each individual ranger, however, has little idea of how their data or even the data from their site fit into the broader patterns identified here.

Did the study involve field work? ☐ Yes ☒ No

Reporting for specific materials, systems and methods

We require information from authors about some types of materials, experimental systems and methods used in many studies. Here, indicate whether each material, system or method listed is relevant to your study. If you are not sure if a list item applies to your research, read the appropriate section before selecting a response.

Materials & experimental systems

n/a	Involved in the study	
☒	☐	Antibodies
☒	☐	Eukaryotic cell lines
☒	☐	Palaeontology
☐	☒	Animals and other organisms
☒	☐	Human research participants
☒	☐	Clinical data

Methods

n/a	Involved in the study	
☒	☐	ChIP-seq
☒	☐	Flow cytometry
☒	☐	MRI-based neuroimaging

Animals and other organisms

Policy information about studies involving animals; ARRIVE guidelines recommended for reporting animal research

Laboratory animals	No lab animals.
Wild animals	Elephant carcasses were recorded upon encounter during regular patrols.
Field-collected samples	No samples were taken from the field.
Ethics oversight	This project runs under the governance of the Monitoring the Illegal Killing of Elephants (MIKE) programme, which itself has statutory responsibilities under the Convention on International Trade in Endangered Species of Wild Fauna and Flora (CITES). The data were collected as mandated by CITES, no further ethical approval has been sought.

Note that full information on the approval of the study protocol must also be provided in the manuscript.
